# Do China’s pilot free trade zones promote green dual-circulation development? Based on the DID model

**DOI:** 10.1371/journal.pone.0281054

**Published:** 2023-03-10

**Authors:** Liuliu Lai, Yanjun Chang

**Affiliations:** School of Economics and Management, Shanghai Institute of Technology, Shanghai, China; Al Akhawayn University in Ifrane, MOROCCO

## Abstract

Accelerating the formation of a green dual-circulation pattern is an essential strategic choice for China to achieve high-quality development. As a vital link for two-way economic and trade cooperation, the pilot free trade zone (PFTZ) is an important window for promoting green dual-circulation development. From the perspective of green dual-circulation, this paper attempts to construct a comprehensive index system of green dual-circulation by entropy weight method based on Chinese provincial panel data from 2007 to 2020 and uses the Propensity Score Matching–Difference in Differences method to test the policy impact of PFTZ building on regional green dual-circulation. The empirical results show that: (1) the establishment of PFTZs significantly promotes regional green dual-circulation development by 3%-4%. This policy effect has a strong positive impact on the eastern regions; (2) PFTZs can improve regional green dual circulation through the effect of green finance, technological progress, and the agglomeration of innovative talents. The mediating effect of green finance and technological progress is more pronounced; (3) The promotion effect of PFTZs is primarily due to the local green circulation effect, with no significant effects on the surrounding areas; and (4) There is a positive policy linkage effect between PFTZs and the Belt and Road Initiative. This study creates the analytical perspective and empirical support for assessing the policy impact of PFTZs and provides management insights for PFTZ policymakers in promoting green dual-circulation development.

## 1. Introduction

Along with the emergence and protracted spread of the COVID-19 pandemic, the global political and economic landscape is changing at an accelerated pace. Localized trade conflicts and technology blockades have added to the external uncertainty of China’s economic development. Currently, China’s economic progress is confronted by two critical obstacles. The first are those related to the environment and ecology. According to the conventional theory of economic development, environmental sustainability and economic growth are mutually exclusive. In other words, economic development can stimulate quick economic growth by committing significant amounts of production resources at the expense of depleting natural resources [1, 2]. In addition to pitting ecological environmental protection against economic development, this development strategy also ignores the ecological carrying capacity. China is currently the world’s largest carbon emitter, accounting for 29.1% of total global CO_2_ emissions [3]. Water resources, air quality, and other ecological problems are prominent. Additionally, pollution is becoming a greater issue.

The second issue is the obstruction of economic growth. Due to the severe resource imbalance at the beginning of reform and opening up, China alters its trade structure and aggressively expanded foreign commerce. Utilizing its advantages in labor and production, China attracts significant amounts of international investment to acquire capital and technology. With continued economic growth and other changes, China accumulates a relatively strong material base. However, in light of China’s mega-scale nature, the development model that primarily relies on external circulation has a limited role in driving economic growth. From the supply-side perspective, China has a significant advantage in medium-technology manufacturing, but there is still a gap with developed countries in high-technology industries. According to the multiregional input-output table prepared by Asian Development Bank, the domestic value added of the United States’ optoelectronic equipment exports in 2019 is 71.45%, compared to China’s value-added of only 29.02%. The absence of core technology continues to increase the risk of uncertainty and instability in the industry chain. Domestic residents are gradually increasing their desire for high-quality goods and services, according to the demand-side perspective. Demand spillover may be the cause of this occurrence. According to figures from the National Bureau of Statistics (https://data.stats.gov.cn/), China’s final consumption expenditures supported economic growth by 65.4% in 2021. Boosting domestic demand and releasing consumption potential could become a new economic growth point. On the other hand, increasing trade tensions between China and the United States have resulted in higher tariffs and investment restrictions. Additionally, the global value chain is being reconfigured faster thanks to the epidemic’s spread and the development of new technologies.

At this stage, building a green dual-circulation development pattern is a crucial step for China to achieve a win-win situation regarding economic growth and environmental protection [4, 5]. Green dual-circulation development (GDC) is a green, low-carbon, sustainable, and high-quality development model that is founded on the principle of ecological priority and creates a dynamic balance between domestic and international markets. Under the green dual-circulation development pattern, China combines the green development direction and promotes the benign interaction between domestic economic circulation and high level open external circulation. This means that China must continue to deepen its openness while also persevering in changing and innovating its institutions and policies [6]. The Pilot Free Trade Zone (PFTZ), as a new highland for national institutional innovation and opening up, not only creates more favorable conditions for connecting domestic and international markets, but also speeds up domestic market reform and continues to deepen global economic and trade cooperation [7]. Therefore, in order to accelerate the construction of green dual-circulation development pattern, vigorously implementing the PFTZ strategy is an inevitable choice for China.

In May 2021, PFTZs and numerous government departments, including the Ministry of Ecology and Environment, jointly released the “Guidelines on Strengthening Ecological Environmental Protection in Pilot Free Trade Zones to Promote High-Quality Development” (see https://www.mee.gov.cn/xxgk2018/xxgk/xxgk03/202105/t20210531_835481.html). The document places a strong emphasis on fully exploiting PFTZ’s function as a link that connects internal and external cycles. It is also suggested that PFTZ aggressively investigate a high-quality, environmentally friendly development model to support the harmonious growth of trade, reciprocal investment, and other areas with the natural environment.

However, more research and investigation are still needed to determine whether the creation of PFTZs produces the green circulation effect. Based on this, from the perspective of green dual-circulation, this paper tries to construct a comprehensive index system of GDC by entropy weight method and builds a quasi-natural experiment based on the construction of PFTZs to test the policy impact on regional GDC by using the multi-period difference-in-differences model.

This study contributes to the existing literature and fills the gap in the following ways. First, the existing literature lacks a quantitative analysis of the level of GDC. Considering internal circulation, external circulation, and green factors, this paper uses the entropy weight method to construct a comprehensive evaluation index system of GDC. Second, although some academics investigate the economic [8–11], trade [12–15], and environmental [16–18] effects of PFTZs, these policy assessments consistently examine economic growth and environmental protection separately, either disregarding or using China’s dual circulation development layout as a backdrop [6]. Therefore, this study selects a new research perspective and focuses on analyzing the impact of PFTZ policies on GDC. Meanwhile, the first five batches of PFTZs are studied to grasp the policy effects as a whole and expand the scope of assessment. Third, three factors—green finance, technological progress, and the agglomeration of innovative talents—are investigated concerning how PFTZs influence regional GDC. This provides data support for the development path of PFTZ construction to promote GDC. Fourthly and lastly, this paper expands the analysis of the policy linkage between PFTZs and the Belt and Road Initiative on GDC. This provides policy insights for the construction of PFTZs to serve the national strategy.

The remainder of the paper is structured as follows: Section 2 presents the policy background, literature review, and mechanism analysis. Section 3 constructs the econometric model and describes the variable data and sources. The empirical component, Section 4, mostly consists of the baseline regression analysis, a series of robustness tests, heterogeneity analysis, and further analysis. Section 5 is the discussion, while Section 6 concludes and draws some policy implications.

## 2. Policy background, literature review, and mechanism analysis

There are three main parts in this section. The policy background for PFTZ building is discussed briefly first. Second, the research literature on PFTZs in academia is reviewed and summarized. Finally, the mechanism of how PFTZs affect GDC is examined.

### 2.1. Policy background

In order to follow the new trend of global economic and trade development, the establishment of PFTZ is a significant initiative for China to build a higher level of the opening platform. This marks a shift in China’s economic goals to a stage of high-quality development [19]. As a testing ground for China to promote reform and opening up, the core function of PFTZ is institutional innovation. PFTZ improves China’s trade freedom, investment facilitation, and financial liberalization through institutional innovation. The results of these reforms will be replicated and extended to the national level after successful trials. Since the construction of Shanghai PFTZ in 2013, China has approved the establishment of six batches of 21 PFTZs in total (Table A1 in S1 Appendix). In terms of spatial layout, China’s PFTZ essentially consists of four different types of seaports, inland ports, airports, and island ports. A new pattern of regional cooperation and open integration is developing.

### 2.2. Literature review

Studies in the literature related to this paper mainly considered three aspects: the economic effects of constructing PFTZs, the environmental impacts arising from economic development driven by PFTZs, and the development trend of foreign trade integration within PFTZs.

Firstly, the economic effects of constructing PFTZs are mainly discussed and studied from the following perspectives. Based at the institutional level, unlike traditional bonded zones or export-processing zones, PFTZs rely on the benefits of institutional innovation to integrate and exploit important production-related elements and further unleash economic performance [8–11]. From the perspective of investment and trade, the construction of PFTZs creates new opportunities for trade and investment facilitation development. On the one hand, PFTZs can transform the conventional foreign trade development model. By reducing trade costs, PFTZs bring long-term economic benefits [12, 13]. On the other hand, PFTZs, through the negative list management model, optimize the investment environment and attract foreign enterprises to invest in the zone [14]. From the perspective of financial innovation, financial liberalization may result from improved financial capital flow made possible by PFTZs [15]. From the perspective of knowledge spillover, PFTZs provide a platform for knowledge sharing, which promotes the exchange of new knowledge and technology among domestic and foreign enterprises. Meanwhile, the accumulation of knowledge within enterprises enables creative technological change and injects new development vitality into economic growth [20, 21].

Secondly, with the promotion of green development, scholars gradually begin to pay attention to environmental effects generated during the process of constructing and developing PFTZs. From the standpoint of sustainable development, [22–24] proposed that port management should balance the relationship between economic prosperity and environmental quality. [25] pointed out that green ports can improve the competitiveness and performance level of ports while reducing environmental pollution. [16] used China (Guangdong) PFTZ to evaluate the influence of creating PFTZs on environmental welfare. The authors’ empirical evidence indicates that PFTZs tend to agglomerate low-end industries and aggravate pollution due to the presence of factors such as a weak technical foundation and a lack of awareness of environmental protection. By assessing the impact of PFTZs on urban green total factor productivity, [17] showed that building PFTZs contributes positively to the health and green performance of cities. [18] examined the green impact of the Shanghai PFTZ and concluded that PFTZ’s green development is essential for fostering high-quality regional development. [26] and [27] reported that PFTZs play a critical role in reducing climate pollution and negative environmental externalities. They suggested that the government could stimulate the sustainable development momentum of ports through a combination of policy measures. To ensure the sustainability of ports, [28–30] argued that port transportation systems must be made more environmentally friendly.

The third aspect is about the development trend of integrating domestic and foreign commerce in PFTZs. The COVID-19 pandemic’s effects made international trade more vulnerable by limiting the flow of people and goods through PFTZs [31, 32]. However, PFTZ’s circular linkage role has not yet been fully exploited in terms of coordinating domestic and international trade [33]. PFTZ’s two-way opening is a crucial development paradigm for satisfying consumer demand [34, 35]. Meanwhile, along with the construction of Belt and Road and the signing of RECP, PFTZ’s present stage of development is focused on encouraging the growth of internal and external circulation [36].

This paper aims to make a significant contribution to the field of PFTZ research. Existing studies give this paper a certain research foundation, although there are still significant gaps. PFTZ is the major platform for connecting two-way markets and a new high ground for institutional innovation, both of which are essential for advancing green dual circulation. The literature lacks an understanding of green dual circulation and how to quantify the level of GDC. Second, it is currently a key strategic step for China to achieve a win-win situation for ecological conservation and high-quality economic development. Prior research consistently distinguished the two and evaluated the policy effects of PFTZs separately. The new dual-circulation development layout, however, is not taken into account or is just seen as the research context. Third, there are certain structural parallels between the Belt and Road Initiative and PFTZs, both of which aim to liberalize trade and investment. The two policies interact and reinforce one another, while the policy linkage impact of PFTZs and the Belt and Road Initiative has not been adequately examined in the available studies.

### 2.3. Mechanism analysis

**Fig 1:**
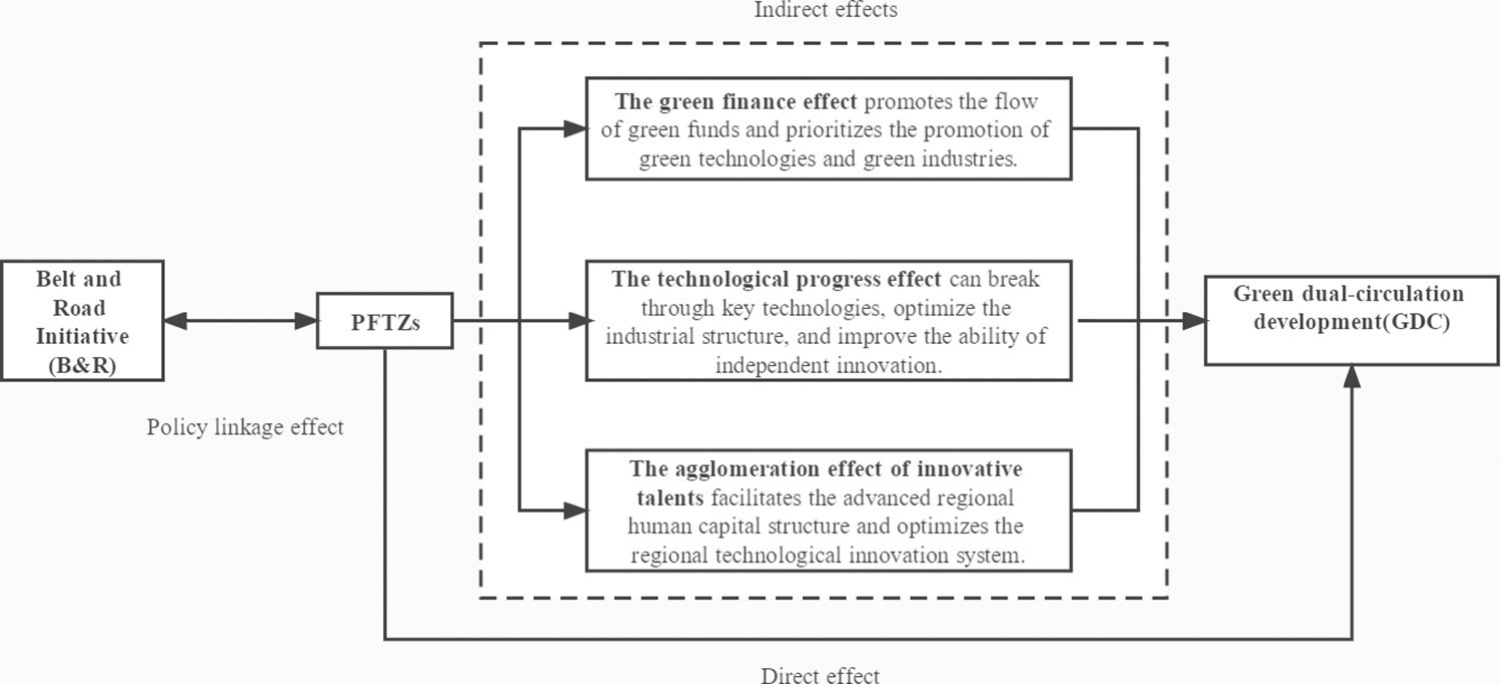
The framework of the influence of PFTZs on GDC. Source: Compiled by the author.

As the new highland for institutional innovation and opening up, PFTZs integrate global quality resources and link domestic and international markets. PFTZs are actively encouraging green dual-circulation growth while also playing up its important hub position. PFTZs can promote GDC by fostering green finance, technological progress, and the agglomeration of innovative talents, as demonstrated in Fig 1.

First, the establishment of PFTZs promotes regional GDC by vigorously promoting green finance. PFTZs’ highly transparent institutional environment provides favorable conditions for the green transformation of the traditional financial structure. In addition, PFTZs’ financial market is more developed and has a high capacity to draw in funds, which supports the free flow of green capital [37]. By directing financial resources to low-pollution and high-efficiency enterprises, PFTZs can effectively improve capital allocation and promote green economic growth.

Second, PFTZs can foster technological innovation and progress, which in turn positively affects GDC. On the one hand, PFTZs enhance businesses’ capacity for independent innovation by maximizing domestic resources and raising R&D spending [38]. PFTZs, on the other hand, introduce high-quality foreign capital, resulting in the acquisition of novel knowledge and technology.

Third, PFTZs effectively draw innovative talent clusters, realizing regional GDC. The creation and growth of PFTZs depend heavily on high-quality innovative talents [39]. By establishing the talent introduction mechanism, PFTZs attract highly qualified innovative talents from both home and abroad, which in turn fosters creativity and innovation. Additionally, the concentration of high-quality innovative talents is conducive to the advanced structure of regional human capital.

## 3. Methodology and data

This section focuses on two aspects of model construction and data selection. Specific definitions of variables and mathematical formulas for models are given in detail.

### 3.1. Methodology

#### 3.1.1. Difference-in-differences (DID) model

DID method is the essential method for assessing the effect of policy implementation. The basic idea of this method is to regard policy implementation as a “quasi-natural experiment”. In this paper, DID model is used to estimate the policy effect of PFTZ construction on the regional green dual circulation. Considering that PFTZs are established in batches, this paper extends the model to the multi-period DID model for policy evaluation. The following benchmark model is constructed:

$${GDC}_{it}=\alpha_{0}+\alpha_{1}{PFTZ}_{it}+\gamma X_{it}+\mu_{i}+\delta_{t}+\varepsilon_{it}$$ (1)

where the subscripts *i* and *t* represent the region and year, respectively; *GDC*_*it*_ is the explained variable, which indicates the level of GDC in region *i* in year *t*; *PFTZ*_*it*_ represents the core explanatory variables; *X*_*it*_ represents a series of control variables, which control other variables affecting GDC including government intervention, per capita gross domestic product, industrial structure, financial scale, and energy structure; *μ*_*i*_ and *δ*_*t*_ represent individual-fixed effects and time-fixed effects, respectively; *ε*_*it*_ is a random error term. This paper focuses on the coefficient *α*_1_ of *PFTZ*, the positive or negative value of which reflects the degree of influence of building PFTZs on regional GDC. If *α*_1_ is significantly positive, this indicates that the construction of PFTZs can contribute to GDC.

In addition, satisfying the parallel trend assumption is a vital premise of DID method. That is, the dependent variables of the treatment and control groups should have the same trend of change before the policy was implemented. Therefore, drawing on the ideas of the event study method [40], the following dynamic test model was constructed.

$${GDC}_{it}=\alpha_{2}+\sum_{k\geq -5}^{5}{\alpha_{k}PFTZ}_{it}^{k}+\gamma X_{it}+\mu_{i}+\delta_{t}+\varepsilon_{it}$$ (2)

where the explained variable *GDC*_*it*_ is identical to Eq (1); ${PFTZ}_{it}^{k}$ represents the *k*-th year in which the treatment group decided to establish PFTZ. *α*_*k*_ is the coefficient that represents the average difference between the experimental and control groups before and after the policy’s implementation. In this paper, the five years before and after the implementation of PFTZs are selected as the time interval under observation.

#### 3.1.2. Propensity score matching (PSM) method

The purpose of this study is to assess the impact of PFTZ construction on the regional green dual-circulation development. That is, this paper aims to reveal whether there is an actual causal relationship between PFTZs and green dual-circulation development. Selection bias, however, frequently causes considerable disturbances to the estimation results in empirical studies. DID method is prone to sample selection row bias, which reduces the reliability of the model results. Therefore, this paper combines PSM method to deal with the differences in characteristics between the experimental and control groups as a way to determine the causal relationship between PFTZs and green dual-circulation development. The theoretical framework of PSM is the “counter-fact inference model”. The basic idea is to find samples from the control group with characteristics similar to those of the treatment group using propensity score values, and then to use differences in the independent variables to explain the differential behavior of the dependent variables.

#### 3.1.3. Mediating effect model

The mediating effect model is constructed to empirically analyze the impact mechanism of PFTZs on GDC.


$$M_{it}=\beta_{0}+\beta_{1}{PFTZ}_{it}+\beta_{2}X_{it}+\mu_{i}+\delta_{t}+\varepsilon_{it}$$ (3)

$${GDC}_{it}=\gamma_{0}+\gamma_{1}{PFTZ}_{it}+\gamma_{2}M_{it}+\mu_{i}+\delta_{t}+\varepsilon_{it}$$ (4)

*M* is the mechanism variable, and it includes three indicators: green finance, independent innovation, and innovative talent. Eq (3) is used to test the impact of PFTZ building on the impact mechanism variable, *M*. If the coefficient of *β*_1_ is significant, this indicates that PFTZs have an impact on the mechanism variable. Then, Eq (4) is based on Eq (1) with the addition of the mediating variable *M*. If *γ*_1_ is significant and smaller than the value of *α*_1_ under the assumption that *β*_1_ is significant, this indicates that there is a mediation effect. That is, the influence of PFTZs on GDC is partly due to the mediation variable. If *γ*_1_ is insignificant and *γ*_2_ is significant, it is a full mediating effect, indicating that PFTZs are fully influenced by the mediating variable on GDC.

### 3.2. Variable selection

#### 3.2.1. Explained variable

Green dual-circulation development (GDC). To overcome the speculative nature of the subjective assignment method, this paper uses the entropy weight method (EWM) to measure the level of GDC. Based on the information entropy of indicators, weights are objectively assigned to the indicator layers. The specific calculation of equations is as follows:

First, each indicator in the index system is normalized using the max-min normalization method:

$$Z_{ij\lambda }=\left\{ \begin{array}{c} \frac{x_{ij\lambda }-x_{min}}{x_{max}-x_{min}}+\alpha ,\;for\;positive\;indicator\\ \frac{x_{max}-x_{ij\lambda }}{x_{max}-x_{min}}+\alpha ,\;for\;inverse\;indicator \end{array}\right.$$ (5)

where *x*_*ijλ*_ denotes the original value of *λ*-th index of *j*-th province in the *i*-th year. *Z*_*ijλ*_ is the normalized index value. To eliminate the influence of the 0 value, add a minimum value (*α* = 0.00001) close to the value of *Z*_*ijλ*_ after the normalization.

Calculate the standardized index value *P*_*ijλ*_:

$$P_{ij\lambda }=\frac{Z_{ij\lambda }}{\sum_{i=1}^{h}\sum_{j=1}^{m}Z_{ij\lambda }}$$ (6)

Calculate the information entropy *E*_*λ*_ of the *λ*-th index:

$$E_{\lambda }=-\frac{\sum_{i=1}^{h}\sum_{j=1}^{m}P_{ij\lambda }lnP_{ij\lambda }}{ln(h\times m)}$$ (7)

Calculate the weight of each index:

$$W_{\lambda }=\frac{1-E_{\lambda }}{\sum_{\lambda =1}^{n}(1-E_{\lambda })}$$ (8)

Finally, calculate the evaluation factor *GDC*_*ij*_ of the *j*-th province in the *i*-th year:

$${GDC}_{ij}=P_{ij\lambda }\times W_{\lambda }$$ (9)

As shown in Table 1, according to the connotation of green dual circulation, this paper constructs the index system of the GDC level from three dimensions: internal circulation, external circulation, and green factors.

**Table 1:** Comprehensive evaluation index system of GDC.

**Dimension Layer**	**Sub-Level**	**Explanation**	**Attributes**	**Weights**
Internal circulation	Production	Ratio of new product sales revenue to main business revenue	+	0.094
	Distribution	Ratio of disposable income per capita to GDP	+	0.050
	Exchange	Ratio of total retail sales of consumer goods to GDP	+	0.035
		Ratio of the added value of transportation, storage, and postal industry to GDP	+	0.046
	Consumption	Ratio of per capita consumption expenditure to per capita GDP	+	0.059
		Final consumption rate	+	0.056
External circulation	Foreign trade dependence	Ratio of total import and export trade to GDP	-	0.012
	Two-way investment	Ratio of foreign direct investment to GDP	+	0.230
		Ratio of outward foreign direct investment to GDP	+	0.272
Green factors	Environmental regulation	Ratio of industrial pollution control investment to the value-added of the secondary industry	+	0.124
	Green patent	Ln (sum of the number of green invention patents and green utility model patents)	+	0.012
	Wastewater	Ratio of total wastewater discharge to GDP	-	0.006
	Waste gas	Ratio of sulfur dioxide emissions to GDP	-	0.005

From the perspective of internal circulation, the production, distribution, exchange, and consumption segments all contribute significantly to the smooth operation of the domestic economy. The development of internal circulation is primarily motivated by the production chain. The ability of the supply side to create new demand is measured by the sales revenue from new products in high-tech fields. Reasonable income distribution is a necessary condition for the healthy development of internal circulation, and it also provides the essential prerequisites for circulation and consumption links. The exchange link, which can affect the distribution and consumption links in addition to the production link, is a crucial medium element of the entire internal circulation process. The total retail sales of consumer products represent the level of commodity circulation. The value added by transportation and other industries assesses the degree of distribution system development. Additionally, consumption upgrading is the key strategy for improving internal circulation. The quality of consumption level is measured through per capita consumption expenditure and regional consumption share.

From the external circulation dimension, it is mainly composed of two parts: foreign trade dependence and two-way investment. The degree to which national or regional economic development is supported by foreign commerce is reflected in its foreign trade dependence. However, overreliance on foreign trade can lead to increased economic vulnerability. The support of two-way investment is essential for improving the standard of GDC. Increased two-way investment has the potential to take advantage of global factor advantages and spur green investment. Cooperation in foreign investment can also maximize resource allocation while raising awareness of green development. Therefore, import and export volume is used to measure foreign trade dependence. Foreign direct investment and outward direct investment represent two-way investment levels.

From the green dimension, environmental regulation facilitates the reduction of green economic losses through market mechanisms. The percentage of investments made in reducing industrial pollution is used in this study to gauge the severity of environmental regulation. Green innovation is crucial to environmental and sustainable development strategies [41, 42]. Thus, the quantity of green patent applications serves as a gauge for green innovation. Wastewater and waste gas are used to measure the level of environmental pollution.

#### 3.2.2. Core explanatory variables

The dummy variable, *PFTZ*, is the core explanatory variable of this paper. Following the implementation of the policy, *PFTZ* = 1 for the provinces creating PFTZs. Other provinces without a PFTZ are given a value of 0. Since the sixth batch of PFTZs (Beijing, Hunan, and Anhui) are established later, this paper temporarily ignores the sixth batch of PFTZs and selects the first five batches of PFTZs as research objects. Due to incomplete data on Tibet, the control group is the remaining provinces without PFTZ. See Table A2 of the S1 Appendix for the sample of the respective treated group and control group.

#### 3.2.3. Mechanism variables

Based on the mechanism analysis, this paper analyzes the influence mechanism from three perspectives: green finance, technological progress, and innovation talent gathering. As a new financial innovation model, green finance combines a variety of financial instruments to direct financiers and investors toward environmental protection [43]. Following He et al. [44], this paper creates the green finance index (GF) using four indicators: green credit, green investment, green insurance, and environmental support. Table A3 of the S1 Appendix displays the relevant indicators and weights presented in the evaluation system. Technological progress (Tech) is measured by the number of patents granted per 10,000 people, which can well reflect independent innovation capacity. In addition, employees in six industries, including scientific research, education, finance, culture and sports, information transmission, and leasing services, are defined as innovative talent. The agglomeration of innovative talent (TG) is calculated using the percentage of innovative talent.

#### 3.2.4. Control variables

For analysis in this study, the following control variables are selected. Government intervention (GI) is measured by the proportion of general budget spending at the regional level to GDP. Per capita gross domestic product (PGDP) represents the level of regional economic development. Industrial structure (IS) is measured by the ratio of the value-added of the tertiary sector to the value-added of the secondary sector. Financial size (Fin) is measured by the balance of deposits and loans of financial institutions as a share of GDP at the end of the year. Energy structure (ES) is measured by the share of coal consumption in total energy consumption. The abbreviations and explanations for all variables are listed in Table 2.

**Table 2:** List of abbreviations and explanations of all variables.

Variable		Abbreviation	Explanation
Explained variable	Green dual-circulation development	GDC	Comprehensive evaluation index system
Core explanatory variable	Pilot free trade zone	PFTZ	Dummy Variable (0 or 1)
Mechanism variables	Green finance	GF	Green finance index
	Technological progress	Tech	Invention patents granted per 10,000 people
	Agglomeration of innovative talents	TG	Total number of employees in the six industries (% of total population)
Control variables	Government intervention	GI	General budget spending (% of GDP)
	Per capita gross domestic product	PGDP	Per capita gross domestic product
	Industrial structure	IS	Ratio of the value-added of the tertiary sector to the value-added of the secondary sector
	Financial size	Fin	The balance of deposits and loans of financial institutions (% of GDP)
	Energy structure	ES	The coal consumption (% of total energy consumption)

### 3.3. Data sources

Due to the incomplete data on Tibet and the exclusion of the sixth batch of PFTZs, the data used in this paper are the panel data of 27 provinces in Mainland China from 2007 to 2020. The relevant data are mainly obtained from the China Statistical Yearbook, the China Environmental Yearbook, and the Statistical Bulletin of China’s Outward Foreign Direct Investment from 2007 to 2020. In terms of green patent acquisition, WIPO’s green technology classification list is used to identify green inventions in the patent database. The descriptive statistics of each variable are shown in Table 3.

**Table 3:** Descriptive statistics.

**Variable**	**Obs**	**Mean**	**Std. Dev.**	**Min**	**Max**
GDC	378	0.158	0.047	0.100	0.440
GI	378	0.255	0.115	0.100	0.760
PGDP	378	4.712	2.594	0.690	15.730
IS	378	1.036	0.418	0.500	3.170
Fin	378	2.942	0.853	1.400	6.260
ES	378	0.965	0.422	0.340	2.500
GF	378	0.158	0.073	0.060	0.420
Tech	378	1.144	1.568	0.040	9.730
Talent	378	0.026	0.010	0.010	0.100

## 4. Empirical analysis

The following five parts—balance test analysis of PSM method, regression analysis, time trend analysis, robustness test, heterogeneity analysis, and further analysis—are presented as the empirical findings of this study.

### 4.1. Balance test analysis of PSM method

**Fig 2:**
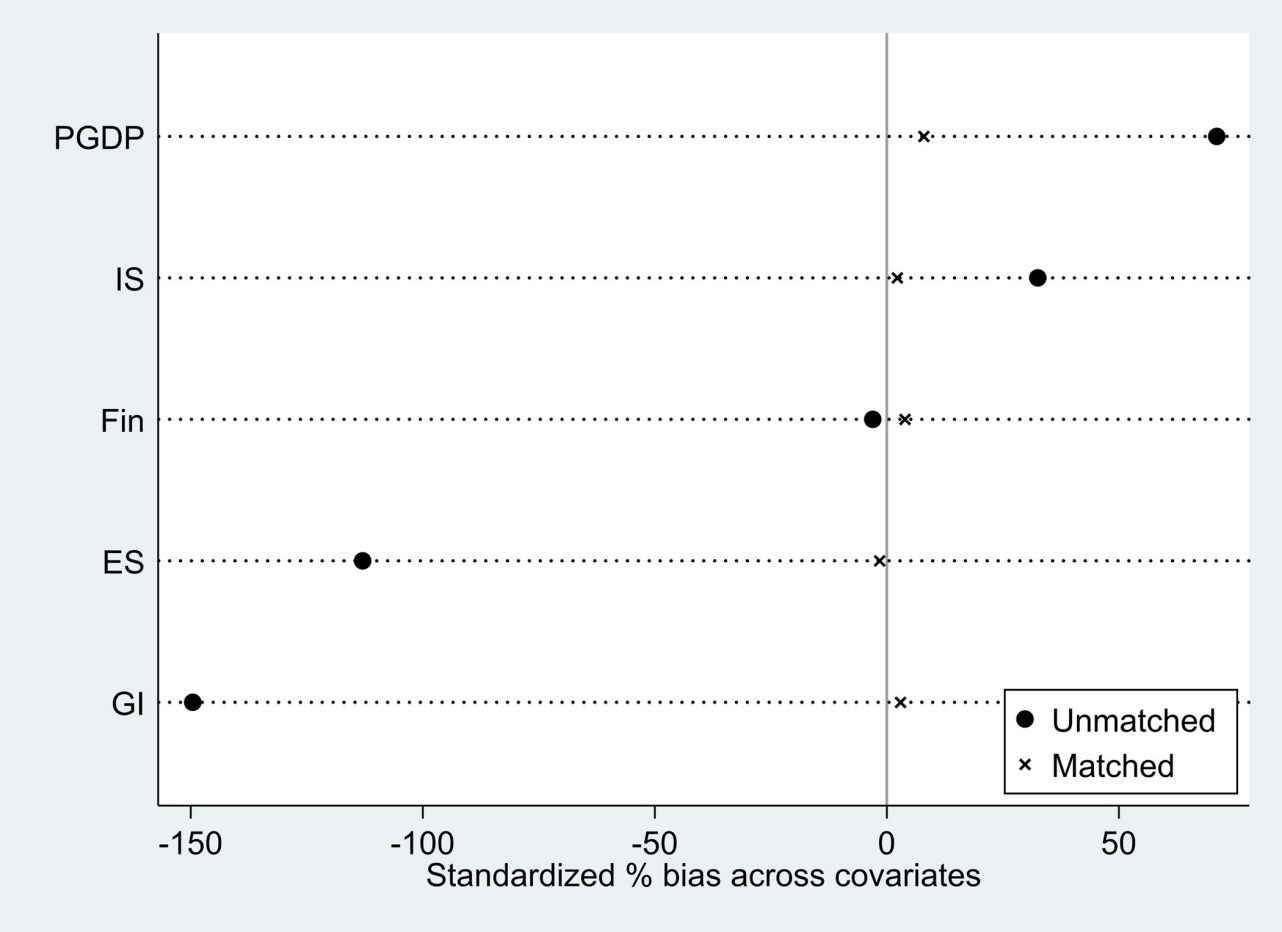
Differences in covariates of variables before and after matching.

Matching balance tests are run on the sample before regression estimation. Referring to Gentzkow [45], this paper uses the kernel density method to estimate logit regression on the sample. The differences in the covariates of the variables before and after matching are shown in Fig 2. The results of the balance test in Table 4 show that the deviation of each covariate is reduced to within 10% after matching. This indicates that there is no significant difference between the treatment and control group covariates after matching. In light of this, the kernel density matching estimation findings are reasonable.

**Table 4:** Balance test results.

Covariate	Unmatched Matched	Mean		%bias	t	p>|t|
		Treated	Control			
GI	U	0.205	0.355	-149.600	-15.190	0.000***
	M	0.244	0.241	2.900	0.380	0.703
PGDP	U	5.254	3.629	71.100	6.00	0.000***
	M	3.396	3.214	8.000	0.840	0.403
IS	U	1.078	0.952	32.500	2.780	0.006***
	M	0.873	0.864	2.200	0.190	0.850
Fin	U	2.934	2.959	-3.000	-0.270	0.784
	M	2.565	2.533	3.900	0.380	0.705
ES	U	0.813	1.268	-113.000	-11.440	0.000***
	M	1.004	1.010	-1.600	-0.150	0.879
	Ps R2		LR chi2		p>chi2	
Unmatched	0.679		326.790		0.000	
Matched	0.004		0.930		0.968	

### 4.2. Regression analysis

According to Eq (1), Table 5 shows the estimated effects of PFTZs on green dual circulation. The fundamental DID estimations are displayed in columns (1) and (2) based on the initial samples. Column (3) and (4) presents the results of PSM-DID estimation following kernel matching. Both regressions employ two-way fixed effects models. As shown in Table 5, the regression results demonstrate that the estimated coefficients of the core explanatory variables are positively significant regardless of whether control variables are included or not, and regardless of whether DID method or PSM-DID method is used. That is, the construction of PFTZs can significantly promote regional green dual-circulation development.

**Table 5:** Regression results.

	DID		PSM-DID	
	(1)	(2)	(3)	(4)
*PFTZ*	0.0315**	0.0275***	0.0366***	0.0430***
	(0.0121)	(0.0080)	(0.0104)	(0.0123)
GI		0.0217	0.0009	0.0082
		(0.0782)	(0.0905)	(0.0862)
Pop		0.0099***	0.0116***	0.0115***
		(0.0030)	(0.0038)	(0.0040)
IS		0.0432**	0.0380	0.0370
		(0.0206)	(0.0338)	(0.0353)
FS		0.0330**	0.0408*	0.0420*
		(0.0145)	(0.0217)	(0.0217)
ES		0.0155	0.0406**	0.0479**
		(0.0147)	(0.0180)	(0.0176)
Constant	0.1526***	-0.0557	-0.1114	-0.1243
	(0.0022)	(0.0596)	(0.0863)	(0.0866)
Benchmark factor ×time trend	No	No	No	Yes
Time fixed effects	Yes	Yes	Yes	Yes
Individual fixed effects	Yes	Yes	Yes	Yes
R-sq	0.6387	0.6995	0.6994	0.7032
N	378	378	260	260

Notes: Standard errors are in parentheses

*, ** and *** indicate significant differences at p < 0.01, p < 0.05 and p < 0.001, respectively.

In fact, the creation of PFTZ typically necessitates taking into account the degree of economic growth, historical mission, and resource availability of each region. These differences over time may have different effects on the level of green dual-circulation development in the region, which could subsequently affect the estimation results. Following [46], this paper adds cross-product-term of the benchmark factors and linear trends over time to control for the influence of these factors. The benchmark factors include whether it was established as a special economic zone in 1979 and whether it was a municipality directly under the central government. From column (4) of Table 5, it can be found that the estimated coefficients of the regression results are still significantly positive at the 1% level.

After mitigating the endogeneity problem caused by the sample selection bias, the results in Table 5 demonstrate that all estimated coefficients are significantly positive at the 1% level and have values between 3% and 4%. Hence, the establishment of PFTZs significantly promotes regional green dual-circulation development by 3%-4%.

### 4.3. Time trend analysis

Table 6 shows related regression results. The estimation results in column (1) show that none of the regression coefficients are significant for the five years before the establishment of PFTZs. This indicates that there is no significant difference between the experimental and control groups in the level of regional green dual-circulation development before the establishment of PFTZs. That is, the requirements of the parallel trend assumption are satisfied. While column (2) shows that the regression coefficients are all significantly positive after the establishment of PFTZs. Meanwhile, PFTZ building has considerably improved the policy’s impact over time.

**Table 6:** Time trend results.

	(1)		(2)
*PFTZ* ^−5^	0.0010	*PFTZ* ^1^	0.0354**
	(0.0050)		(0.0151)
*PFTZ* ^−4^	-0.0046	*PFTZ* ^2^	0.0374*
	(0.0071)		(0.0208)
*PFTZ* ^−3^	-0.0032	*PFTZ* ^3^	0.0476***
	(0.0094)		(0.0153)
*PFTZ* ^−2^	-0.0072	*PFTZ* ^4^	0.0418**
	(0.0116)		(0.0196)
*PFTZ* ^−1^	0.0130	*PFTZ* ^5^	0.0431**
	(0.0131)		(0.0172)
Controls	Yes		Yes
Year fixed effects	Yes		Yes
Province fixed effects	Yes		Yes

Notes: Standard errors are in parentheses

*, ** and *** indicate significant differences at p < 0.01, p < 0.05 and p < 0.001, respectively.

### 4.4. Robustness tests

#### 4.4.1. Placebo test

**Fig 3:**
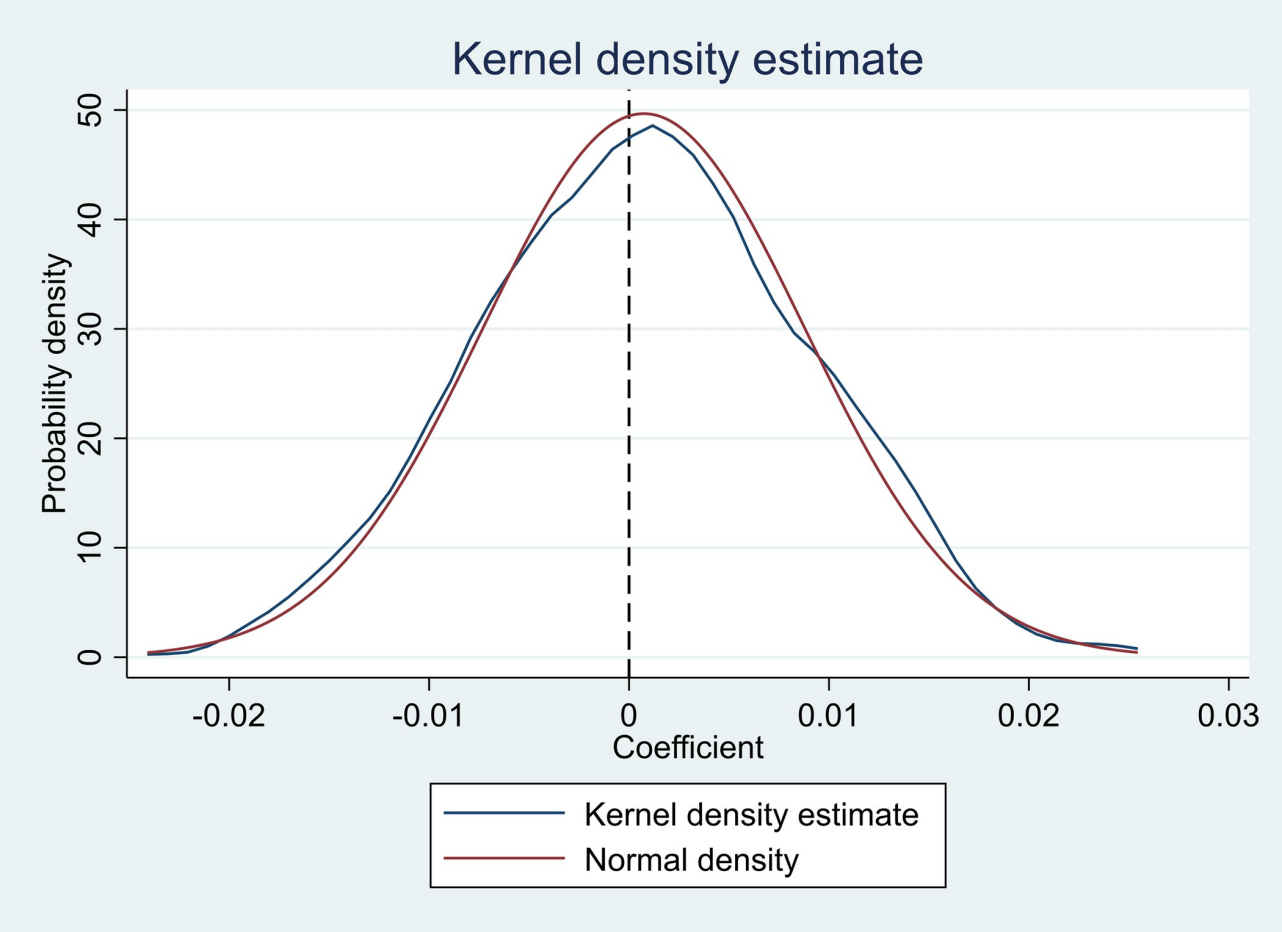
Placebo test.

To avoid the effect of unobservable factors, this paper adopts the placebo test in the sample by randomly selecting the pilot provinces of PFTZs and generating the policy time. The placebo test randomly chose 18 unduplicated pilot provinces from the sample to serve as the pseudo-treatment group to maintain the same number of provinces in the treatment group. The year of policy implementation for the pseudo-treatment group is also randomly assigned. Based on this, the coefficient estimate is obtained by regressing the newly generated pseudo-policy dummy variable. The distribution of the obtained coefficients was presented in Fig 3 after the above process was repeated 500 times.

Fig 3 shows that the mean of all estimated coefficients obtained from the 500 random samples was close to zero. In the placebo test, the real estimates in Table 5 are also notable outliers. Thus, it can be concluded that the baseline regression results in this paper are not due to some unobservable factors.

#### 4.4.2. Expected effects test

The construction of PFTZs has the potential to be expected well in advance of the official implementation of the policy. These expected effects may confound the study’s findings. To exclude expected effects, this paper adds the dummy variable *PFTZ*_*t*−1_ to the benchmark regression model. It denotes the year before the implementation of PFTZ. The regression results are displayed in column (1) of Table 7, where the coefficient of *PFTZ*_*t*−1_ is not statistically significant, while the PFTZ coefficient is still significantly positive. As a result, once the assumption of the expected effects is met, the promotion effect of PFTZs creation on regional green dual-circulation development exists.

**Table 7:** Robustness tests.

	(1)	(2)	(3)	(4)
	Expected effect	Excluding the impact of other policies		
		LCP	B&R	GFP
PFTZ	0.0430***	0.0362***	0.0366***	0.0368***
	(0.0123)	(0.0107)	(0.0105)	(0.0103)
*PFTZ*_*t*−1_	0.0187			
	(0.0155)			
lcp		0.0070		
		(0.0135)		
b&r			0.0006	
			(0.0122)	
gfp				0.0033
				(0.0108)
Controls	Yes	Yes	Yes	Yes
Time fixed effects	Yes	Yes	Yes	Yes
Individual fixed effects	Yes	Yes	Yes	Yes
Constant	-0.1243	-0.1192	-0.1109	-0.1101
	(0.0866)	(0.0906)	(0.0852)	(0.0861)
R-sq	0.7032	0.6999	0.6994	0.6996
N	260	260	260	260

Notes: Standard errors are in parentheses

*** indicates significant differences at p < 0.001.

#### 4.4.3. Excluding the impact of other policies

Other regional policies that are contemporaneous or functionally similar may also interfere with the study results. According to compiling other significant policies before and after the construction of PFTZs, this paper speculates that the pilot low-carbon (LCP) policy in 2010 [47, 48], the alignment of provinces along the Belt and Road Initiative (B&R) in 2013 [49], and the green finance innovation policy (GFP) in 2017 [50] may affect the development of the regional green dual-circulation development. Therefore, this paper adds dummy variables of these three policies to the control variables of the baseline regression model (Eq (1)).

The regression results are presented in columns (2)-(4) of Table 7. After controlling for these three policies, the regression coefficient of PFTZ remains positively significant at the 1% level. This demonstrates that other policies have not interfered with the green circulation effect of PFTZs. The test also lays the groundwork for the following study to explore the policy linkage effect of PFTZs and the Belt and Road Initiative.

### 4.5. Heterogeneity analysis

One of the key responsibilities of creating the green dual-circulation growth pattern is promoting coordinated regional development. Currently, China’s PFTZs achieve full coverage from the eastern region to the western region. The effects of PFTZs in various regions may differ based on variances in geographic location and level of market development. This study divides the sample into eastern and central-western areas by location to further examine the heterogeneous effects of PFTZs on China’s regional green dual-circulation development.

Table 8 shows that, in comparison to the central-western regions, the establishment of PFTZs has a stronger role in promoting green dual-circulation development in the eastern regions. The geographical dominance and relative development of the eastern areas may be contributing factors to this outcome. Physical capital, institutional elements, and local government spending all contribute to the formation of a thriving environment for two-way trade and investment as well as the agglomeration of innovative talents, which is better suited to advancing green dual circulation development.

**Table 8:** Heterogeneity analysis.

	(1)	(2)
	Eastern	Central-western
PFTZ	0.0250*	0.0115
	(0.0147)	(0.0160)
Controls	Yes	Yes
Time fixed effects	Yes	Yes
Individual fixed effects	Yes	Yes
Constant	-0.1023	0.1089
	(0.2339)	(0.1351)
R-sq	0.7526	0.6891
N	127	115

Notes: Standard errors are in parentheses

* indicates significant differences at p < 0.01.

### 4.6. Further analysis

To enrich the research findings, this paper further analysis of PFTZ policy’s impact mechanism, its effects on nearby regions, and its linkages to the Belt and Road Initiative.

#### 4.6.1. Transmission mechanism analysis

Based on previous mechanism analysis, this paper introduces three mediating variables, namely, green finance, independent innovation, and innovative talents, respectively, to test the influence mechanism.

The odd-numbered columns of Table 9 show the estimation results based on Eq (3) with the mediating variables as the explained variables, respectively. It can be seen that the estimated coefficients of *PFTZ* were all significantly positive at the 1% confidence level. This means that the construction of PFTZs significantly promotes green financial innovation and technological progress, and attracts the agglomeration of innovative talents. The even-numbered columns show that the estimated coefficients of PFTZ are all positively significant. The mediating factors’ estimated coefficients are all positive at the 1% level as well. Meanwhile, the Sobel test p-value for the mediating effect was less than 0.05, indicating that the mediating effect holds. Specifically, green finance, technological progress, and the agglomeration of innovative talents contribute to the partially intermediary role in boosting green dual circulation in PFTZs, with the indirect effect accounting for 35.52%, 68.62% and 64.05% respectively. Thus, PFTZs can improve regional green dual circulation through the effect of green finance, technological progress, and the agglomeration of innovative talents. Among them, the mediating effect of technological progress and the agglomeration of innovative talents is more pronounced.

**Table 9:** Results of transmission mechanism test.

	(1)	(2)	(3)	(4)	(5)	(6)
	GF	GDC	Tech	GDC	Talent	GDC
*PFTZ*	0.0155***	0.0230**	0.3721***	0.0210***	0.0020**	0.0254***
	(0.0055)	(0.0087)	(0.1337)	(0.0071)	(0.0009)	(0.0079)
GF		0.2848**				
		(0.1219)				
Tech				0.0175***		
				(0.0050)		
Talent						0.9981*
						(0.5179)
Controls	Yes	Yes	Yes	Yes	Yes	Yes
Time fixed effects	Yes	Yes	Yes	Yes	Yes	Yes
Individual fixed effects	Yes	Yes	Yes	Yes	Yes	Yes
Constant	0.0678***	-0.0751	-5.3557***	0.0378	-0.0170	-0.039
	(0.0236)	(0.0578)	(1.1379)	(0.0485)	(0.0122)	(0.0572)
Sobel Z	2.419**		4.969***		5.934***	
	(0.0048)		(0.0045)		(0.0035)	
Proportion of indirect effect	0.3552		0.6862		0.6405	
R-sq	0.9694	0.7053	0.9340	0.7215	0.8746	0.7051
N	378	378	378	378	378	378

Notes: Standard errors are in parentheses

*, ** and *** indicate significant differences at p < 0.01, p < 0.05 and p < 0.001, respectively.

#### 4.6.2. Radiation effect or siphon effect

The benchmark regression analysis shows that the construction of PFTZs can effectively promote local green dual-circulation development. However, the policy effect on neighboring provinces cannot be determined yet. This paper further investigates whether there is a radiation effect or siphoning effect of the establishment of PFTZs on green dual-circulation development of the surrounding areas. Referring to [51], the model is constructed as follows.

$${GDC}_{it}=\alpha_{0}+\alpha_{2}{near}_{c}\times {nearpost}_{t}+\gamma X_{it}+\mu_{i}+\delta_{t}+\varepsilon_{it}$$ (10)

*near*_*c*_ is a region dummy variable that takes the value of 0 or 1. *near*_*c*_ = 1 denotes the neighboring province c where PFTZ is built; conversely, *near*_*c*_ = 0. *nearpost*_*t*_ denotes the time dummy variable. If PFTZ has established around province c after *t*-th year, then *nearpost*_*t*_ = 1; otherwise, the value is assigned to 0. The model’s additional variables and parameters are identical to those in the model (1). *α*_2_ is the test of whether PFTZ produces the radiative or siphon effect. If *α*_2_ is significantly positive, this indicates that the establishment of PFTZ can drive green dual-circulation development of neighboring provinces and produce a radiation effect. If *α*_2_ is significantly negative, this indicates that PFTZ has a siphoning effect on the neighboring regions. It should be noted that this model mainly examines the effect of PFTZs on neighboring provinces. So, the model estimation does not include the sample of PFTZs itself.

The estimation results of model (10) are reported in Table 10. The results show that the construction of PFTZs forms a negative but insignificant spillover effect on green dual circulation in the neighboring provinces. This implies that the siphoning effect of the construction of PFTZs on the neighboring provinces has not yet appeared. Additionally, this demonstrates that the development of PFTZs encourages regional green dual-circulation development mostly through the local areas’ green circulation effect, which may have a negative impact on the surrounding areas.

**Table 10:** The radiation effect or siphon effect of PFTZs.

	(1)	(2)
*near*_*i*_×*nearpost*_*t*_	-0.0240*	-0.0097
	(0.0105)	(0.0101)
Controls	No	Yes
Time fixed effects	Yes	Yes
Individual fixed effects	Yes	Yes
Constant	0.1633***	0.0088
	(0.0028)	(0.1254)
R-sq	0.6468	0.7122
N	126	126

Notes: Standard errors are in parentheses

* and *** indicate significant differences at p < 0.01 and p < 0.001, respectively.

#### 4.6.3. Policy linkage analysis

The construction of PFTZs and the Belt and Road Initiative together provide the essential strategic platform for China’s higher level opening up. The two are similar in function and support each other. Based on this, this paper further explores the policy linkage effect. By introducing the indicators of the docking provinces along the Belt and Road initiative, this paper extends the benchmark model (1) and constructs a triple difference model for testing. The specific difference model setting was as follows:

$${GDC}_{it}=\alpha_{0}+\alpha_{1}{PFTZ}_{it}\times {road}_{i}+\gamma X_{it}+\mu_{i}+\delta_{t}+\varepsilon_{it}$$ (11)

In Eq (11), *road*_*i*_ is a dummy variable, and if region *i* is the province or city along the Belt and Road Initiative, set *road* = 1; otherwise, *road* = 0. Table 11 displays the specific estimation results.

**Table 11:** Regression results of policy linkage effects.

	(1)	(2)
PFTZ*road	0.0246*	0.0171**
	(0.0155)	(0.0083)
Controls	No	Yes
Time fixed effects	Yes	Yes
Individual fixed effects	Yes	Yes
Constant	0.1554***	-0.0491
	(0.0018)	(0.0559)
R-sq	0.6255	0.6881
N	378	378

Notes: Standard errors are in parentheses

*, ** and *** indicate significant differences at p < 0.01, p < 0.05 and p < 0.001, respectively.

The regression results show that the estimated coefficients of PFTZ*road are all significantly positive. This indicates that there is a significant positive policy linkage effect between PFTZs and the Belt and Road Initiative.

## 5. Discussion

As a crucial node linking domestic and international markets, PFTZ construction is of great significance to high-quality circulation development of China’s economy. Currently, most policy studies on the establishment of PFTZs segregate environmental protection from economic growth and examine the policy effects of PFTZs only from a single dimension. These studies ignore the importance of the dynamic balance between economic growth and environmental protection. Green dual-circulation is a crucial tactic for China, though, to deal with shifting internal and external problems and to achieve win-win development regarding economic growth and environmental protection. To fill this gap and enrich the literature on PFTZs, this paper uses the multi-period DID method to test the policy impact of the establishment of PFTZs on regional green dual-circulation development.

From the empirical perspective, the benchmark regression’s findings show that the establishment of PFTZs promotes regional green dual-circulation development by 3%-4%. The results passed various tests such as the placebo test, balance test and robustness test. This ensured the randomization of the experimental group and the validity of the study findings. Meanwhile, the research results support the conclusions of [17, 18, 26] to a certain extent that the establishment of PFTZs has beneficial environmental benefits. Additionally, there is regional heterogeneity in this policy effect. Compared with the central-western regions, PFTZs in the eastern regions are more effective in promoting green dual-circulation development.

To enrich the findings, this paper further explores the impact mechanism of PFTZs to promote green dual-circulation development. The empirical results reveal that PFTZs can improve regional green dual circulation through the effect of green finance, technological progress, and the agglomeration of innovative talents. Among them, the mediating effect of technological progress and the agglomeration of innovative talents is more pronounced. Second, the paper further examines the policy implications of PFTZs on neighboring regions. The regression results indicate that PFTZs have a siphoning effect on the neighboring areas. This result confirms the findings of [52]. Finally, and importantly, this paper finds that there is a positive policy linkage effect between PFTZs and Belt and Road Initiative on green dual-circulation development, which provides empirical support for PFTZs construction to serve national strategies.

## 6. Conclusions

Building the green dual-circulation pattern is the essential path for China to achieve high-quality economic development. The green dual-circulation pattern calls for constructing a greater scope and higher level of openness to promote sustainable connectivity and interaction between domestic and international. Therefore, as the new high ground for China’s institutional innovation and opening up, PFTZ plays an essential pivotal role in promoting green dual circulation. The findings of this paper provide appropriate strategic paths for the construction of PFTZs to support green dual-circulation development, as well as the following management insights for decision-makers, strategic planners, and government agencies.

1. PFTZs should always be centered on institutional innovation. Transform internal and external circulation of development through deep reform and innovation to solve fundamental problems. Meanwhile, the management committee of PFTZs should receive more reform autonomy from the government. By supporting systematical reforms in the green field, PFTZs will eventually narrow the gap with international advanced opening rules and improve the business environment to achieve high-quality green dual circulation.
2. Given the heterogeneous characteristics, PFTZs should implement differentiated policy orientation. Relying on the resource and location advantages of different regions, PFTZs must overcome the traits of development convergence to achieve coordinated and sustained regional development. For instance, one of the key tasks of the Guangdong PFTZ is to support the integrated economic growth of the mainland and the Guangdong-Hong Kong-Macao Greater Bay Area. Taking advantage of the two sides of the Pearl River Estuary, the Guangdong PFTZ has accumulated a good economic foundation and formed a complete industrial system and broad consumer market.
3. The primary drivers of green dual-circulation development are technological progress and innovative talents. By developing an innovation platform for industry-university-research cooperation, PFTZs should cooperate with a variety of innovation forces and foster the autonomous innovative vitality of innovation subjects. PFTZs should also enhance the creation of the talent ecological system as the source of technical innovation. PFTZs can improve the treatment of innovative talents and smooth the flow of innovative elements by implementing talent programs. Additionally, PFTZs should focus on regional cooperation to avoid the policy siphon effect. By reducing local protectionism and environmental regulatory differences, PFTZs can play a positive leading role and generate positive spillover effects, thereby promoting regional coordinated green development.
4. The empirical results confirm the significant policy linkage effect of PFTZs and the Belt and Road Initiative. Thus, PFTZs should adhere to a higher level of openness and deeply collaborate with the Belt and Road Initiative. Relying on the Belt and Road Strategy, PFTZs should fully release the dividends of institutional innovation and create a free and open environment for both domestic and foreign businesses. The strategic partnership can also strengthen the green economy and trade cooperation between China and surrounding nations or areas.

This study may have the following limitations. First, the development of green dual-circulation has not been adequately studied quantitatively in the literature. The evaluation method for GDC is built from three dimensions in this research, while the indicators might not have been chosen with enough refinement and comprehensiveness. Future research can more effectively determine the degree of GDC by adding more precise variable elements. Second, while the research sample for this paper—Chinese provincial data—obtains adequate and reliable data, there is a dearth of empirical support at the micro-firm level. In the future, we will consider collecting more detailed panel data of PFTZs’ listed companies to examine the impact of PFTZs on green dual-circulation development from a microscopic perspective. In addition, this paper uses DID method to help estimate the average treatment effect of PFTZs on regional green dual-circulation development. However, in terms of spatial dimension, DID model may not be accurately identified. In future research, we will construct the spatial differences-in-differences (SDID) model to measure the policy effects comprehensively to precisely identify spatial spillover effects.

## Supporting information

S1 Appendix (DOCX)
