# Supplementary material for: Do China’s pilot free trade zones promote green dual-circulation development? Based on the DID model
Source: PLoS One. 2023 Mar 10;18(3):e0281054. doi: 10.1371/journal.pone.0281054 (PMC10004592; doi:10.1371/journal.pone.0281054)
Supplement: S1 Appendix — (DOCX) [file pone.0281054.s001.docx]

Appendix A

See Table A1 to Table A3.

**Table A1. Construction of China’s Pilot Free Trade Zone.**

| **Function positioning** | **Implementation time** | **Province** | **Specific Goals and Roles** |
| --- | --- | --- | --- |
| The experimental field for institutional innovation and reform to promote financial, trade and investment liberalization | The first batch  (September 2013) | Shanghai | Demonstration leadership |
|  | The second batch  (April 2015) | Guangdong | Trade and investment facilitation to serve the mainland |
|  |  | Tianjin | Developing modern service industries |
|  |  | Fujian | Deepening cross-strait economic cooperation |
|  | The third batch  (March 2017) | Liaoning | Industrial base structural adjustment |
|  |  | Zhejiang | Developing international commodity trade liberalization |
|  |  | Henan | Serving the Belt and Road to build a modern integrated transportation hub |
|  |  | Hubei | Strategic emerging industries and high technology base |
|  |  | Chongqing | Serving the Western Development Strategy |
|  |  | Sichuan | Inland open economy highland |
|  |  | Shaanxi | Serving the Belt and Road Strategy |
|  | The fourth batch  (October 2018) | Hainan | Free trade port with Chinese characteristics |
|  | The fifth batch  (August 2019) | Shandong | Developing the marine economy |
|  |  | Jiangsu | Focusing on innovative development and industrial transformation |
|  |  | Guangxi | ASEAN cooperation and trade Center |
|  |  | Hebei | New types of industrialization base |
|  |  | Yunnan | Cross-border cooperation center |
|  |  | Heilongjiang | The essential window for opening up to the north |
|  | The sixth batch  (September 2020) | Beijing | Developing service trade |
|  |  | Anhui | Developing digital economy and trade |
|  |  | Hunan | Service Central Rise Strategy and Manufacturing innovation heights |

**Table A2. Experimental samples.**

| **Treated Group** | **Control Group** |
| --- | --- |
| Shanghai (2013); Guangdong, Fujian, Tianjin (2015); Liaoning, Zhejiang, Henan, Hubei, Chongqing, Sichuan, Shannxi (2017); Hainan (2018); Shandong, Jiangsu, Guangxi, Hebei, Yunnan, Heilongjiang (2019) | Shanxi, Inner Mongolia; Jilin; Jiangxi; Guizhou; Gansu; Qinghai; Ningxia; Xinjiang |

**Table A3. The comprehensive evaluation system of green finance.**

| **1st-class index** | **2st-class index** |
| --- | --- |
| Green credit  (45%) | Ratio of interest expenses for energy-intensive industrial industries |
| Green investment  (25%) | Ratio of environmental pollution control investment to GDP |
| Green insurance  (15%) | Ratio of agricultural insurance income |
| Environmental support  (15%) | Ratio of financial environmental protection expenditure |
